# Acute and subacute IL-1β administrations differentially modulate neuroimmune and neurotrophic systems: possible implications for neuroprotection and neurodegeneration

**DOI:** 10.1186/1742-2094-10-59

**Published:** 2013-05-07

**Authors:** Cai Song, Ye Zhang, Yilong Dong

**Affiliations:** 1 Research Institute of Marine Drug and Nutrition, Guangdong Ocean University, Zhanjiang, Guangdong, China; 2 Graduate Institute of Neural and Cognitive Sciences, China Medical University & Hospital, Taichung, Taiwan; 3 School of Life Science, No.2 Cuihu Bei Road, Kunming, Yunnan, 650091, China; 4 Department of Biomedical Science, University of Prince Edward Island, 550 University Ave, Charlottetown, C1A 4P3, Canada

**Keywords:** Acute and 8 day repeated IL-1β administration, Microglia, Astrocytes, Memory, Neurotrophins, Neurotrophin receptors, Cytokines

## Abstract

**Background:**

In Alzheimer’s disease, stroke and brain injuries, activated microglia can release proinflammatory cytokines, such as interleukin (IL)-1β. These cytokines may change astrocyte and neurotrophin functions, which influences neuronal survival and induces apoptosis. However, the interaction between neuroinflammation and neurotrophin functions in different brain conditions is unknown. The present study hypothesized that acute and subacute elevated IL-1β differentially modulates glial and neurotrophin functions, which are related to their role in neuroprotection and neurodegeneration.

**Method:**

Rats were i.c.v. injected with saline or IL-1β for 1 or 8 days and tested in a radial maze. mRNA and protein expressions of glial cell markers, neurotrophins, neurotrophin receptors, β-amyloid precursor protein (APP) and the concentrations of pro- and anti-inflammatory cytokines were measured in the hippocampus.

**Results:**

When compared to controls, memory deficits were found 4 days after IL-1 administrations, however the deficits were attenuated by IL-1 receptor antagonist (RA). Subacute IL-1 administrations increased expressions of APP, microglial active marker CD11b, and p75 neurotrophin receptor, and the concentration of tumor necrosis factor (TNF)-α and IL-1β, but decreased expressions of astrocyte active marker glial fibrillary acidic protein (GFAP), brain-derived neurotrophic factor (BDNF) and TrK B. By contrast, up-regulations of NGF, BDNF and TrK B expressions were found after acute IL-1 administration, which are associated with the increase in both glial marker expressions and IL-10 concentrations. However, TrK A was down-regulated by acute and up-regulated by subacute IL-1 administrations. Subacute IL-1-induced changes in the glial activities, cytokine concentrations and expressions of BDNF and p75 were reversed by IL-1RA treatment.

**Conclusion:**

These results indicate that acute and subacute IL-1 administrations induce different changes toward neuroprotection after acute IL-1 administrations but neurodegeneration after subacute ones.

## Background

In the last two decades, many studies have found that neuroinflammation is causally related to the onset and progress of several neurodegenerative disorders, including Alzheimer’s disease (AD). Increased microglial activity and pro-inflammatory cytokine releases may contribute to neuronal dysfunction and death in neurodegenerative diseases [1,2]. Among many inflammatory triggers and mediators, both experimental and clinical data have suggested IL-1β as the most potent pro-inflammatory cytokine in neurodegeneration. First, a genetic study has shown that the inheritance of a specific IL-1 gene polymorphism is associated with an earlier age of AD onset and increases the risk for AD development by as much as six-fold [3]. Second, IL-1β cultured with cortical neurons has been found to increase mRNA expressions and the concentration of amyloid precursor proteins (APP), neuropathological markers of AD [3,4]. Third, our team has previously reported that subacute administration of IL-1β to rats can reduce acetylcholine (ACh) release and impair memory [5]. Therefore, IL-1 administration to rodents has been popularly used as a model for studying the interaction between inflammation, brain functions, and memory deficits in neurodegenerative and psychiatric diseases [6,7].

However, neuroinflammation has two sides; it may be beneficial in promoting homeostasis and neuron survival, but can also result in tissue injury through the over-action of inflammatory mediators. Even though many studies have reported that increased IL-1 release contributes to neurodegeneration in both acute and chronic brain conditions, findings from acute ischemia, stroke and brain injuries show that the release of IL-1 and other pro-inflammatory cytokines, such as TNF-α, may protect neurons [8-12]. However, many studies have demonstrated that blocking IL-1 or reducing inflammation could reduce neuron death and symptoms of disability as well as save patients’ lives [13]. This conflict raises important questions: whether and how acute and subacute or chronic IL-1 releases differentially modulate central nervous system (CNS) functions, which may result in opposite effects during different brain conditions. To answer this question, the pro- and anti-inflammatory mechanisms and the neuroprotective system in the brain should be compared in acute and 8-day repeated IL-1-induced models.

It is well known that in different brain diseases, both activated microglia and astrocytes can produce pro-inflammatory, anti-inflammatory and neurotrophic factors. However, microglia may be more involved in the inflammatory response in neuropathological conditions, while astrocytes may have more supporting and maintaining functions, including releasing neurotrophins and anti-inflammatory cytokines. Neurotrophins and their receptors compose a major neuroprotective system in the brain because they stabilize and maintain homeostasis (protection and repair), clean up neurotoxins, regulate neurotransmission and modulate neuronal regenesis and degenesis [14-17]. The most often studied neurotrophins related to neurodegeneration are nerve growth factor (NGF) and brain-derived neurotrophic factor (BDNF). The highest mRNA levels of these neurotrophins have been found in the hippocampus, which indicates their important roles in neuroendocrine and cognitive behavior [18]. Indeed, our research group previously reported that the down-regulation of NGF mRNA expression is correlated with the reduction of ACh release after IL-administration for 8 days [5].

BDNF also plays a crucial role in cognition, learning, and memory formation by modulating synaptic plasticity and is, therefore, a critical molecule in dementia and neurodegenerative diseases. Two types of neurotrophin receptors have already been found: tyrosine kinases (TrK A, B, C) and low-affinity NGF (p75) receptor [18]. The receptor for NGF is TrK A and the receptor for BNDF is TrK B, while both neurotrophins also combine with p75 receptors. The binding of neurotrophins to their TrK receptors causes signaling events, which promote neuron survival, whereas the activation of the p75 pathway may trigger apoptosis or enhance the selectivity of neurotrophin binding for specific TrKs [19,20]. Hence, neurotrophins may activate different cellular mechanisms depending on which type of receptors they interact with.

Recently, increasing evidence has suggested that neuroinflammation may trigger neuroprotection or neurodegeneration through the neurotrophic system [21]. Increased and decreased expressions or concentrations of neurotrophins have been reported in acute and chronic neurodegenerative diseases. For example, both IL-1 and NGF concentrations were markedly increased in the frontal cortex within 24 hours of traumatic brain injuries [22], and BDNF was found to be increased in acute spinal cord injury [23-25]. In the brain of AD patients, increased or decreased NGF and BNDF mRNA or protein expressions have been reported [18,26,27]. Furthermore, TrK A and TrK B receptors have been shown to be down-regulated in AD brains [18]. However, it is unclear whether and how acute and subacute glial activation and IL-1 released in response to these different conditions are related to changes in neurotrophin systems. The present study hypothesized that acute and 8-day repeated IL-1 administrations differentially modulate the activity of microglia and astrocytes, which may change the inflammatory and neurotrophin systems, thereby providing protective or degenerative effects on brain functions such as memory and Aβ deposition. To demonstrate this hypothesis, the present study evaluated 1) the effects of 1-day (acute) and 8-day (subacute) IL-1 administrations on hippocampus-dependent working memory in rats, and 2) the gene and protein expressions of the microglial active marker CD11b and astrocyte marker glial fibrillary acidic protein (GFAP), APP, neurotrophins and receptors, and their relationship with pro- and anti-inflammatory cytokines in the hippocampus.

## Materials and methods

### Animals

Male Long-Evans rats (250 to 280 g, Charles River, Quebec, Canada) were housed in pairs and maintained under a 12-h light, 12-h dark cycle, room temperature at 21 ± 1°C, with food and water available *ad libitum*. There were two independent experiments (EXP1 and EXP2). In EXP1, rats were divided into four groups of 10 as: 1) acute saline treatment (1 day); 2) acute IL-1 (1 day); 3) subacute saline (8 days) and 4) subacute IL-1 (8 days). In EXP2, rats were divided into two groups of 10 as: 1) subacute IL-1 (8 days) + saline, and 2) subacute IL-1 (8 days) + IL-1 receptor antagonist (RA). Since we previously showed that IL-1RA alone did not affect rat memory and NGF expression [5], we did not study the effects of IL-1RA in the control group. All procedures were reviewed and approved by the Institutional Animal Care and Use Committee in the University of Prince Edward Island, in accordance with the guidelines of the Canadian Council on Animal Care (06–004, 1001966). The experimental design for EXP1 is presented in Diagram 1. The experimental procedure for EXP2 was the same as the EXP1.

### Surgery

Following habituation for 1 week, animals were anesthetized with ketamine (100 mg/kg) and xylazine (20 mg/kg) and placed in stereotaxic apparatus. For intracerebroventricular (i.c.v.) injections, guide cannulae (24-gauge) were located stereotaxically over the right lateral ventricles (anterior/posterior=1 mm; medial/lateral=1.6 mm; 1 mm depth) as described in detail elsewhere [28]. A dummy cannula was screwed into the guide cannula to maintain patency. The guide cannula protruding from a concentric custom-made Derelin pedestal was secured to the skull with three screws and dental cement. Tetracycline powder was used to treat the wound. Animals were allowed to recover for 10 days.

### Behavioral training and testing

Foraging tests were conducted on an eight-arm radial maze, consisting of an octagonal center platform (51 cm in diameter, arm-to-arm) connected to eight equally spaced arms (40 × 13 cm). Ten days after the surgery, rats were food deprived to 90% of their free-feeding weight and the delayed spatial win-shift version of the radial maze task was adapted from Song *et al*. [28]. Each trial consisted of a training phase and a memory test phase, separated by a delay (from 5 minutes to 50 minutes). Before the training phase, four arms were randomly baited with food pellets (Bioserv, French, NJ, USA). The other arms were blocked. In the training phase, each rat was allowed 5 minutes to retrieve the food from the four open arms. During the memory test phase, all arms were opened and rats were allowed to explore the maze until they had retrieved food located in the four arms that were blocked during training, or until 5 minutes had elapsed. An arm entry was defined as movement along the arm to the food cup. Errors were recorded as rat entries into unbaited arms. Criterion performance during the memory tests was defined as five or fewer arm entries to locate four food pellets. When criterion performance at a 50-minute delay was maintained for two consecutive days, saline or IL-1 was i.c.v.-injected immediately after the training phase. Since our previous two studies demonstrated that saline or IL-1 administration before the training phase did not significantly impair animal learning [5,28], in the present study injections were only carried out after the training phase for comparing acute and subacute effects. At 45 minutes after the injection, animal error entries were scored at memory test phases. For the acute injection groups, animal behavior was tested only once after saline or IL-1 administration and animals were decapitated on day 2. Controls that received saline injection for 2 days were compared to the group treated with IL-1 for 2 days; the controls that received saline injection for 3 days were compared to the group treated with IL-1 for 3 days, and so on for 8 days.

### I.c.v. injections of IL-1β and IL-1RA

Rat recombinant IL-1 and IL-1RA were obtained from R&D system Inc (Minneapolis, USA) and dissolved in sterile, pyrogen-free saline at doses of 15 ng/10 μL/rat for IL-1 and 100 ng/5 μL/rat for IL-1RA [5]. The dose for IL-1β was based on those used in our previous study (5, 10, and 50 ng) and on other studies (10 to 20 ng) [29,30]. The method for IL-1 and IL-1RA injection was the same as previously described [28]. In brief, rats were gently handled for 2 weeks before the first injection. On the injection day, 10 μL IL-1, IL-1+IL-1RA or saline was drawn into an internal needle that was connected to a micro-injector through a PE 50 polyethylene tube. IL-1, IL-1RA or saline was slowly infused into the brain over a period of 60 s. The injection needle was allowed to remain inside the guide cannula for 1 minute. Rats were returned to their home cages for a 45-minute period before behavioral tests [28].

### Determination of cannula locations

After decapitation, brains were rapidly removed and placed on an ice block. The location of the cannula and the injection site was confirmed in the coronal section of the brain under a microscope. The hippocampuses were dissected quickly on ice and then frozen in N_2_. Animals with injection sites and probes outside of the lateral ventricular were excluded from the data.

### Quantitative PCR

In EXP1, mRNA expressions of CD11b, GFAP, NGF, BDNF, TrK A, TrK B, p75 and APP were measured by quantitative PCR in the hippocampus. The primer sequences for these genes and the internal control, β-actin (Sigma, Canada), are listed in Table 1. The total RNA was purified from brain tissues using RNeasy Mini Kit (Qiagen). cDNA was synthesized from 2 μg total RNA using the Ominiscript RT kit (Qiagen). Primer sequences were purchased from Invitrogen (Carlsbad, CA, USA). The real-time PCR reaction solution contained a final volume of 15 μl QuantiTect SYBR Green PCR Mixture providing a 2.5 mM MgCl_2_, 1pM each of the primers and 0.5 μl cDNA templates. The real-time PCR was optimized to run with conditions of the initial activation at 95°C for 15 minutes, denaturation at 94°C for 15 s, annealing at 55°C for 20 s, and extension at 72°C for 15 s with a single fluorescence measurement and up to 40 cycles. The specificity of the primers was validated by agarose gel electrophoresis and real-time PCR to ascertain that no non-specific products formed prior to the sample analysis. The values were normalized against the internal control, β-actin, since we previously tried GAPDH to compare β-actin expressions in the hippocampus, and did not find a significant difference between these two house-keeping (HK) genes in the IL-1-induced model.

**Table 1:** Primer sequences of quantitative PCR

**Rat genes**	**Primer sequences**
CD11b	5^′^-CAAGGAGTGTGTTTGCGTGT
	5^′^-AGAAGGCTCGGACAACTGAG
GFAP	5^′^-CCAAGATGAAACCAACCT
	5^′^-CGCTGTGAGGTCTGGCTT
NGF-β1	5^′^---CAACAGGACTCACAGGAGCA
	5^′^---GTCCGTGGCTGTGGTCTTAT
BDNF	5^′^---CAAAAGGCCAACTGAAGC
	5^′^---CGCCAGCCAATTCTCTTT
TrK A	5^′^---GGCTTATGCTTGCTGGTC
	5^′^---CTGGGTCAATGCTGTTAGGT
TrK B	5^′^---CACACACAGGGCTCCTTA
	5^′^---AGTGGTGGTCTGAGGTTGG
p75	5^′^---TGCTCCATTTCCATCTCAG
	5^′^---GATAGGTCCGTAATCCTCTTC
APP	5^′^---AAGGGCATCGCTTACAAA
	5^′^---AACCAGCCAGTGACCATC
Actin	5^′^---GTCGTACCACTGGCATTGTG
	5^′^---CTCTCAGCTGTGGTGGTGAA

### Western blot

The rabbit origin antibodies for CD11 (160 KDa), GFAP (50 KDa), BDNF (14 KDa), TrK A (140 KDa), TrK B (95–145 KDa) and p75 (75 KDa) were purchased from Santa Cruz Biotechnology, USA. The samples were spun down at 5,000 rpm for 5 minutes following hippocampus removal and homogenizing in the Teen buffer. The pellets were lysed in the lysis buffer, and then spun down at 13,000 rpm for 10 minutes. After adding sample buffer and boiling, the supernatant was collected and loaded to SDS PAGE gels. After running the gels, the proteins were transferred to nitrocellulose membranes. The proteins were then blotted with specific primary and secondary antibodies. Proteins on the membranes were detected by enhanced chemiluminescence (ECL) and then developed with image film. The bands were scanned and densitometrically analyzed using an automatic image analysis system (NIH Image 1.61 software). All target proteins were quantified by normalizing them to β-actin re-probed on the same membrane and then calculated as a percentage of the control group.

### Measurement of NGF concentration by ELISA kit

β-NGF concentrations were measured in the hippocampus by the ELISA kit, NGF Emaxtm ImmunoAssay System number G7631 (R&D System, Promega, USA) following the instructions provided by the manufacturer. The brain tissues were homogenized with ultrasonication in an extraction buffer (Tris-acetate 20 mM, pH 7.5, NaCl 150 mM, ethylenediamine-tetraacetic acid (EDTA) 1 mM, EGTA 1 mM, sodium-pyrophosphate 2.5 mM, ortovanadate 1 mM, β-glycerolphosphate 1 mM, NaF 100 mM, phenylmethylsulfonyl fluoride (PMSF) 1 mM, leupeptin 1 μg/mL) and centrifuged at 4°C for 20 minutes, 10,000 g, and supernatants were recovered with the solution (EDTA, PMSF). Briefly, 96-well immunoplates were coated with 100 μL per well of polyclonal anti-NGF antibody. After an overnight incubation at 4°C, the plates were washed once with wash buffer (Tris–HCl 20 mM, pH 7.6, NaCl 150 mM, 0.05% Tween^®^ 20) and then blocked for 1 h with the block & sample 1x buffer provided by the manufacturer (200 μL/well). After washing, the samples were incubated in the coated wells (100 μL each) for 6 h at room temperature with shaking. After an additional five washes the immobilized antigen was incubated with an anti-NGF monoclonal antibody overnight at 4°C. The plates were washed again with wash buffer, and then incubated with an anti-rat IgG horseradish peroxidase (HRP) conjugate (100 μL/well) for 2.5 h at room temperature. After another washing, the plates were incubated with a tetramethyl benzidine (TMB)/peroxidase substrate solution for 15 minutes (100 μL/well), provided by the manufacturer). Reactions were then stopped with 100 μL/well 1 N HCl. The colorimetric reaction product was measured at 450 nm using a microplate reader (Dynatech MR 5000, PBI International, USA). NGF concentrations were determined from the regression line for the NGF standard (ranging from 7.8 to 500 pg/mL purified NGF) incubated under similar conditions in each assay. Under these conditions the recovery of NGF in our assay was >90%. The NGF sensitivity of the assay was about 3 pg/g of wet tissue, and cross-reactivity with other related neurotrophic factors (BDNF, neurotrophin-3 and neurotrophin-4) was less than 3%. Data are represented as ng/mL supernatant and all assays were performed in duplicate.

### Measurement of cytokine concentrations by Bio-Plex protein array system

TNF-α, IL-1β and IL-10 were measured in the hippocampus as previously described [31]. In brief, a customized 3-plex rat cytokine panel consisting of fluorescent beads for IL-1β, TNF-α and IL-10 (BioRad, CA, USA) was analyzed with a Luminex protein suspension array system (Bioplex 200, Biorad, CA, USA) according to manufacturer’s instructions. Due to the large binding surface of the beads, this assay is highly sensitive and has been proven before to work well for detecting cytokines from brain tissues [32]. The results were expressed as pg/mL of brain supernatant.

### Data analysis

The behavioral results were analyzed by two-way analysis of variance (ANOVA); duration (acute or subacute) × treatment (saline or IL-1), with repeated measurement for EXP1 and one-way ANOVA for EXP2. Results for mRNA and protein expressions of glial markers, neurotrophins and their receptors and cytokine concentrations were analyzed by two-way (duration × treatment) ANOVA for EXP1 and one-way ANOVA for EXP2. The statistics were expressed by F (=variance of the group means / mean of the within group variances) with the degree freedom. When *P* was <0.05 on ANOVA, the difference between groups was assessed by Newman-Keuls post hoc test (GB-STAT, Dynamic Microsystems, Inc., USA). Significance was set at *P* <0.05. Results are expressed as mean ± standard error of the mean (SEM).

## Results

### Differential effects of acute and subacute IL-1 B administration on working memory

**Figure 1:**
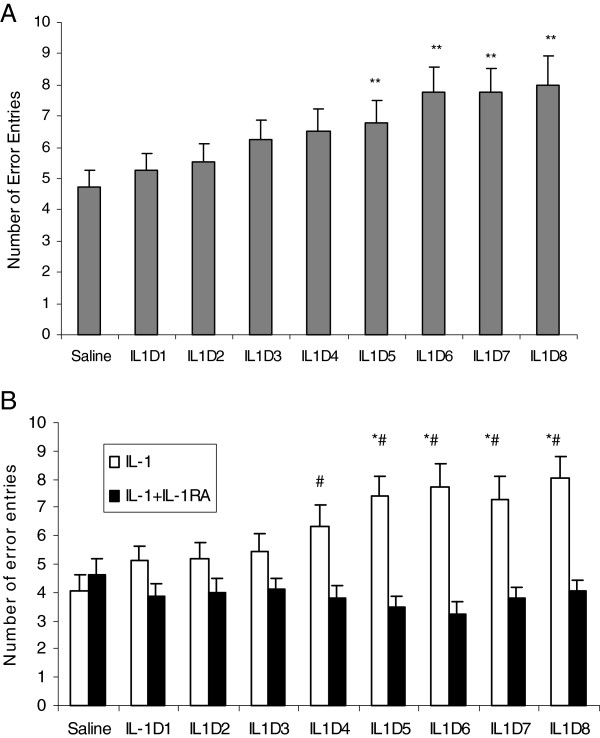
**IL-1-induced changes in the number of entry error in 8-arm radial maze during testing phase. **Before the training, 8 arms were baited with a single 45-mg food pellet at the cup in the end of each arm. In the training phase, four arms chosen randomly were opened, and the remaining arms were blocked. Each rat was allowed 5 minutes to retrieve the food from the four open arms. During the memory test phase, all arms were opened and rats were allowed to explore the maze until they had retrieved food located in the four arms that were blocked during training, or until 5 minutes had elapsed. Errors were recorded as entries into unbaited arms. (**A**) On day 5, subacute IL-1 significantly increased the error number, ^**^P <0.01 versus saline-treated group (n = 7). (**B**) IL-1 receptor antagonist (IL-1RA) reversed subacute IL-1-induced changes in memory retrieval, ^*^P <0.01 versus saline group (n = 8), ^#^*P *<0.01 versus IL-1 group (n = 8).

Rats that received saline i.c.v. administration prior to either the memory training or the testing phase of the delayed working memory task were able to find the location of food pellets in specific arms on the maze. These rats were also able to utilize this information after a 50-minute delay to select and enter a subset of four arms that contained food (Figure 1, training A). The i.c.v. administration of IL-1β prior to training had no effect on the time taken or the number of arm choices to find food rewards during the training period. However, two-way ANOVA confirmed that the treatment factor significantly affected the number of error entries during the memory testing from day 5 to day 8 (day 5, F3,26 = 23.96, *P* <0.001; day 6, F3,26 =14.16, *P* <0.001; day 7, F3,26 = 21.34, *P* <0.001; day 8, F3,26 = 22.19, *P* <0.001). The *post hoc* test confirmed that IL-1 administrations significantly increased the number of error entries during testing phases on days 5, 6, 7 and 8 when compared to the control group (*P* <0.01) (Figure 1A). 8 days repeated IL-1-induced memory impairment was reversed by IL-1RA treatment (day 5, F1,18 = 7.53, *P* <0.05; day 6, F1,18 = 8.61, *P* <0.01; day 7, F1,18 = 10.32, *P* <0.01; day 8, F1,18 = 8.74, *P* <0.01) (Figure 1B).

### IL-1β administration-induced changes in CD11b and GFAP expressions

**Figure 2:**
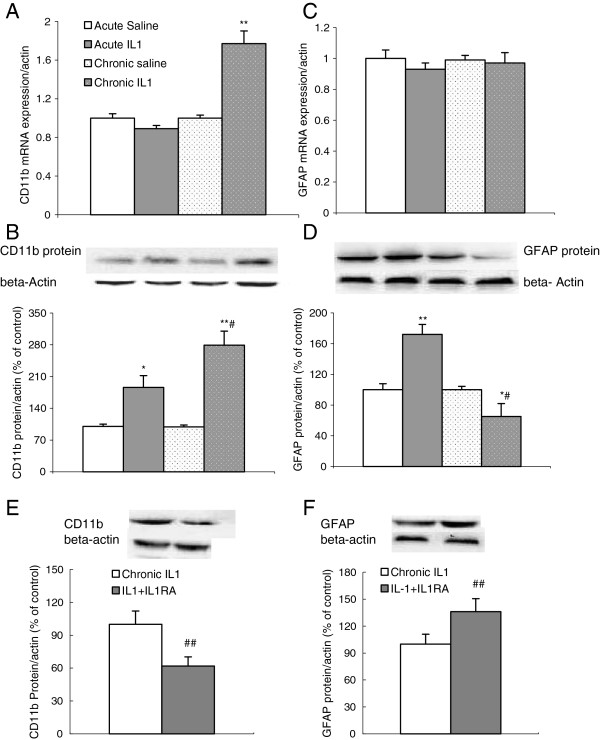
**Effects of acute and subacute IL-1β administrations on mRNA and protein expressions of microglial marker CD 11b and astrocyte marker glial fibrillary acidic protein **(**GFAP), and the effect of IL-1RA treatment on subacute IL-1-induced changes in the rat hippocampus. **(**A**) CD 11b mRNA expressions; (**B **and **E**) CD 11b protein expressions; (**C**) GFAP mRNA expressions; (**D **and **F**) GFAP protein expressions; ^*^P <0.05; ^**^P <0.01 versus the matched control group, ^#^*P *<0.05 versus acute IL-1 group; ^##^*P *<0.01 versus the subacute IL-1 group (mRNA n = 9 to 10; protein n = 7 to 8).

Two-way ANOVA showed that both treatment and duration factors significantly changed gene expressions of the microglial marker CD 11b (treatment: F1,27 = 13.07, *P* <0.001; duration F1,27 = 7.48, *P* <0.05). The post hoc revealed that significant up-regulation of CD11b mRNA expression occurred in the group receiving 8 days of repeated IL-1 administration (*P* <0.01) (Figure 2A). At the protein level, the ANOVA also indicated that the treatment and duration factors had significant effects on CD11b expressions (treatment: F1,27 = 41.5, *P* <0.0001; duration F1,27 = 5.1, *P* <0.05) and that the interaction between these two factors was also significant (F1, 27 = 4.95, *P* <0.05). The post hoc test revealed that IL-1 significantly increased CD11b expression after both acute (*P* <0.05) and subacute (*P* <0.0001) administrations, whereas the increase was much greater in the group that received subacute IL-1 injections (*P* <0.01) (Figure 2B).

With regards to astrocyte activation, two-way ANOVA showed that the treatment factor significantly affected GFAP protein but not mRNA expressions (treatment: F1,27 = 23.46, *P* <0.001) (Figure 2C). The post hoc test demonstrated that acute IL-1 significantly increased, while 8 days repeated IL-1 decreased GFAP protein expressions (*P* <0.01) (Figure 2D).

Results from EXP2 demonstrated that IL-1RA treatment for 8 days significantly attenuated subacute IL-1-induced increase in CD11b and decreased GFAP expressions at the protein level (mRNA expressions were not measured); CD11b: F1,18 = 9.91, *P* <0.01; GFAP: F1,18 = 7.72, *P* <0.01) (Figure 2E and F).

### IL-1β administration-induced changes in the NGF and TrK A system

**Figure 3:**
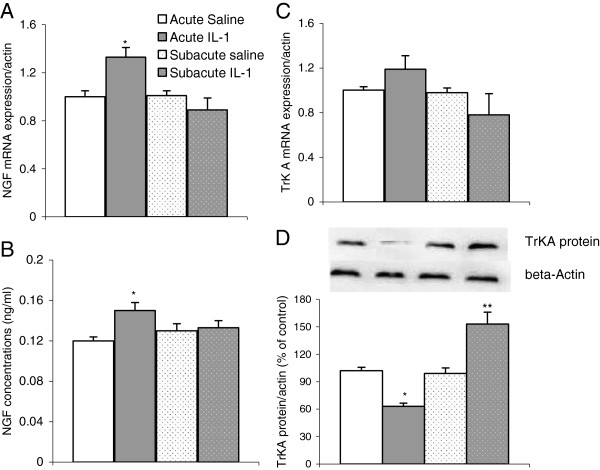
**Effects of acute and subacute IL-1β administrations on mRNA and protein expressions of neurotrophin nerve growth factor and its receptor tyrosine kinase A in the rat hippocampus. **(**A**) Nerve growth factor (NGF) mRNA expressions; **B**, NGF concentrations; (**C**) tyrosine kinase (TrK)A mRNA expressions and (**D**) TrK A protein expressions. ^*^P <0.05; ^**^P <0.01 versus the matched control group (mRNA n = 9 to 10; protein n = 7).

Two-way ANOVA demonstrated that the factor of treatment significantly changed NGF mRNA expressions and concentrations in the hippocampus (mRNA: F1,38 = 7.2, *P* <0.01; concentration: F1,27 = 6.57, p<0.05). The *post hoc* test showed that acute IL-1 administration significantly up-regulated NGF mRNA expressions and increased NGF concentrations (*P* <0.05) (Figure 3A and B). However, 8 days repeated IL-1β administration did not significantly change NGF mRNA expressions and concentrations (Figure 3B).

With regards to the mRNA expressions of NGF receptor TrK A, the ANOVA indicated that neither of two factors had any significant effect on TrK A gene expressions (Figure 3C). However, at the protein level, the treatment factor significantly changed protein expressions (F1,38 = 24.08, *P* <0.0001), and the interaction between the treatment and duration was also significant (mRNA F1,33 = 10.34, *P* <0.01; protein: F1,27 = 9.59, *P* <0.001). The *post hoc* test revealed that acute IL-1 administrations markedly down-regulated TrK A protein expressions (*P* <0.01) (Figure 3D). By contrast, 8 days repeated IL-1 administrations up-regulated this receptor expression (*P* <0.01) (Figure 3D).

### IL-1β administration-induced changes in the BDNF and TrK B system

**Figure 4:**
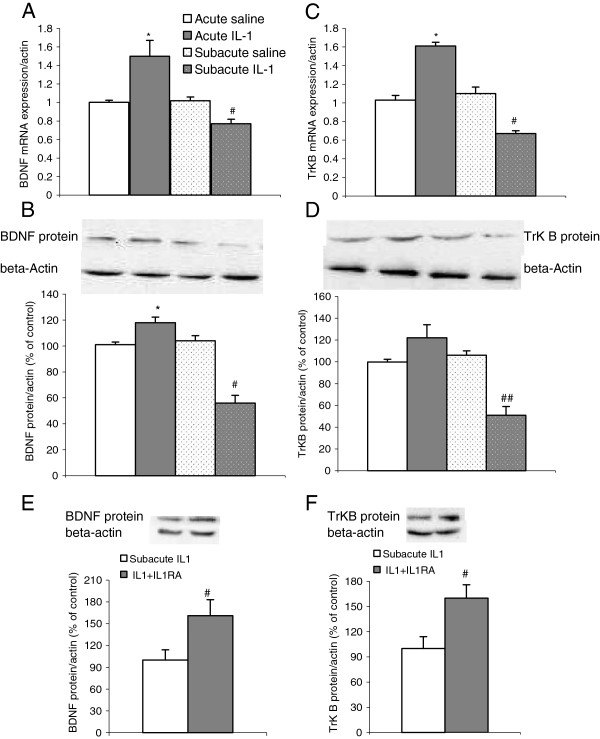
**Effects of acute and subacute IL-1β administrations on mRNA and protein expressions of brain-derived neurotrophic factor and its receptor tyrosine kinase in the rat hippocampus. **(**A**) Brain-derived neurotrophic factor (BDNF) mRNA expressions; (**B **and **E**) BDNF protein expressions; (**C**) Tyrosine kinase (TrK) B mRNA expressions and (**D **and **F**) TrK B protein expressions. ^*^P <0.05 versus acute control group; ^#^*P *<0.05, ^##^*P *<0.01 versus subacute control group and acute IL-1 group (**A**-**D**); ^#^*P* <0.05 versus subacute IL-1 group (**E**,**F**) (mRNA n = 9 to 10; protein n = 7 to 8).

Two-way ANOVA indicated that the treatment factor significantly changed BDNF gene and protein expressions (mRNA: F1,38 = 8.71, *P* <0.001; protein: F1,27 = 15.91, *P* <0.001), and there was a significant interaction between these two factors (mRNA: F1,38 = 13.62, *P* <0.01; protein: F1,27 = 17.97, *P* <0.001). The *post hoc* test indicated that acute IL-1β administration significantly increased BDNF mRNA expressions (*P* <0.05), while 8 days repeated IL-1 reduced BDNF expressions (*P* <0.05) (Figure 4A). Protein expressions of BDNF were not significantly changed by acute IL-1 administration, whereas, consistent with the gene results, a significant reduction in BDNF protein levels was found in the group with subacute IL-1 administrations (*P* <0.05) (Figure 4B). This BDNF reduction induced by 8 days repeated IL-1RA treatment significantly attenuated IL-1-induced decrease in BDNF protein expressions (F1,18 = 13.62, *P* <0.01) (Figure 4E). The IL-1 factor also affected TrK B expressions significantly at both mRNA and protein levels (mRNA: F1,38 = 8.14, *P* <0.01; protein: F1,27 = 15.97, *P* <0.0001) as revealed by two-way ANOVA. The *post hoc* test revealed a similar pattern of changes in mRNA and protein expressions of BDNF between groups. Thus, gene expressions of TrK B were up-regulated after acute IL-1 administration (*P* <0.05) (Figure 4C), but markedly down-regulated by 8 days repeated IL-1 administration (*P* <0.05) (Figure 4C). At the protein level, the increase in TrK B did not reach significance after acute IL-1 treatment, but a significant reduction of TrK B protein level was found after 8 days repeated IL-1 administration (*P* <0.01) (Figure 4D). One way ANOVA demonstrated that IL-1RA treatment significantly reversed the TrK B expressions at the protein level (F1,18 = 13.75, *P* <0.01) (Figure 4f).

### IL-1β administrations-induced changes in the expressions of p75 receptor and APP

**Figure 5:**
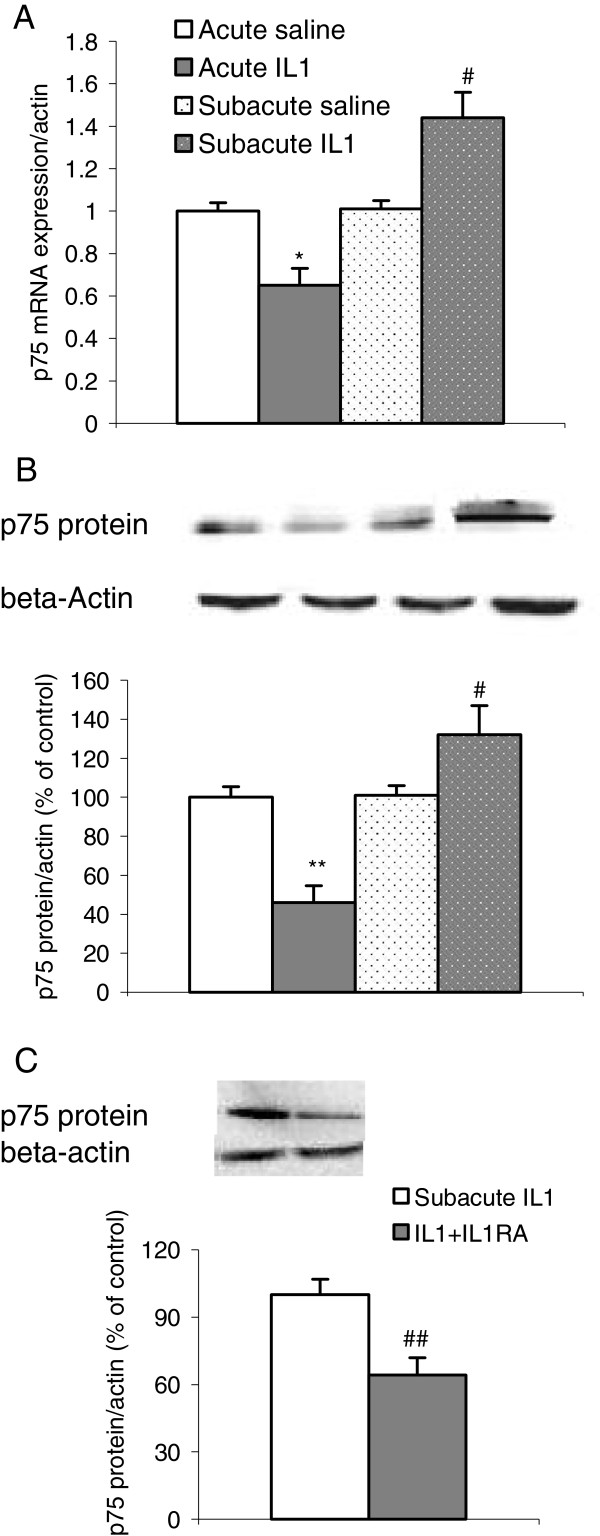
**Effects of acute and subacute IL-1β administrations on mRNA and protein expressions of p75 receptor in the rat hippocampus. **(**A**) p75 mRNA expressions; (**B **and **C**) p75 protein expressions. ^*^P <0.05, ^**^P <0.01 versus acute control group; ^#^*P *<0.05, ^##^*P *< 0.01 versus control group and acute IL-1 group (**A**,**B**); ^##^*P *< 0.01 versus subacute IL-1 group (**C**) (mRNA n = 9 to 10; protein n = 7 to 8).

As shown by Figure 4, the gene and protein expressions of p75 receptor were changed significantly by the treatment (mRNA: F1,38 = 25.34, *P* <0.0001; protein: F1,27 = 20.07, *P* <0.001), as indicated by two-way ANOVA. The interaction between IL-1/saline treatment and duration was also significant (mRNA: F1,38 = 23.54, *P* <0.0001; protein: F1,27 = 21.93, *P* <0.0001). The post hoc test demonstrated that acute and subacute administration of IL-1β exerted opposite effects on p75 receptors at gene and protein levels; acute IL-1 down-regulated mRNA and protein expressions of p75 receptors (*P* <0.01), while 8 days repeated IL-1 significantly increased both expressions (*P* <0.01 and 0.05, respectively) (Figure 5A and B). In the IL-1RA treated group, the increased in p75 protein expression was significantly lower when compared to subacute IL-1 group (F1,18 = 7.92, *P* <0.01) (Figure 5C).

**Figure 6:**
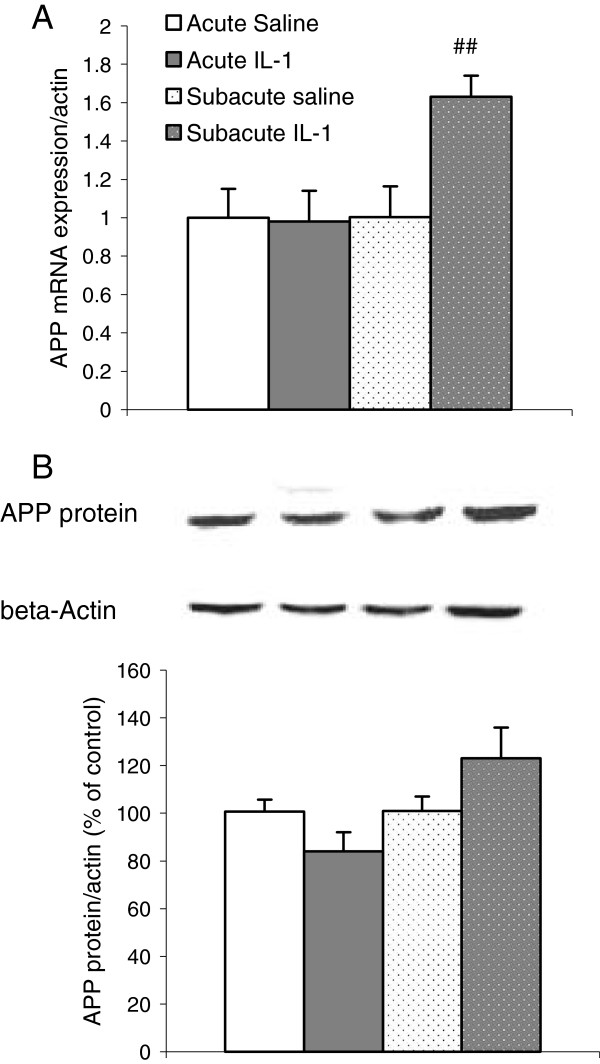
**Effects of acute and subacute IL-1β administrations on mRNA and protein expressions of amyloid precursor protein in the rat hippocampus. **(**A**) Amyloid precursor protein (APP) mRNA expressions; (**B**) APP protein expressions; ^##^*P *<0.01 versus subacute control group and acute IL-1 group (mRNA n = 9 to 10; protein n = 7).

APP gene expressions were shown to be markedly affected by both treatment and duration factors (treatment: F1,38 = 13.02, *P* <0.01; duration: F1,38 = 10.98, *P* <0.01). The interaction between these two factors was also significant (F1,38 = 13.13, *P* <0.001). The *post hoc* test revealed that there was no significant change in APP mRNA expressions in the group with acute IL-1 administration. When compared to the group repeatedly treated with saline for 8 days, a significantly up-regulation of APP mRNA expressions was found in the hippocampal tissues in the animals with 8 days repeated IL-1 administration (*P* <0.01) (Figure 6A). On the other hand, the effect of IL-1 on APP protein expressions only approached significance (F1,27 = 4.33, *P* = 0.051), and the interaction between IL-1 and treatment duration is also only close to significant (F1,27 = 4.23, *P* = 0.054). However, the *post hoc* test did not show any significant changes between groups (Figure 6B).

### IL-1 administration-induced changes in hippocampal IL-1β, TNF-α and IL-10 concentrations

The effects of the treatment factor on hippocampal IL-1β, TNF-α and IL-10 concentrations were significant (IL-1: F1,31 = 7.28, *P* <0.01; TNF: F1,31 = 8.64, *P* <0.01; IL-10: F1,31 = 7.14, *P* <0.01). The effects of treatment duration on these three cytokines were also significant (IL-1: F1,31 = 6.37, *P* <0.05; TNF: F1,31 = 9.72, *P* <0.01; IL-10: F1,31 = 8.52, *P* <0.01). As shown by Table 2, acute IL-1 administration did not change IL-1β concentrations but significantly increased TNF-α and IL-10 concentrations (*P* <0.01), while 8 days repeated IL-1 injection significantly increased both IL-1β and TNF-α but reduced IL-10 levels (*P* <0.05) when compared to the matched saline treatment group (Table 2). IL-1RA treatment significantly reduced IL-1 (F1,18 = 9.36, *P* <0.01) and TNF-α (F1,18 = 7.81, *P* <0.01) concentrations; IL-1 (IL-1 group: 4,125.79 ± 513.36, IL-1 + IL-1RA group: 2,082.53 ± 410.21) and TNF-α (IL-1 group: 1376.72 ± 226.81, IL-1 + IL-1RA group: 395.421 ± 52.39).

**Table 2:** **IL-1**β **administration-induced changes in concentrations of pro-inflammatory and anti-inflammatory cytokines in the hippocampus**

	**Acute saline administration**	**Acute IL-1 administration**	**Subacute saline administration**	**Subacute IL-1 administration**
IL-1β	1579.3 ± 187.2	2016.3 ± 245.2	1523.53 ± 248.52	4072.07 ± 496.04^b^
TNF-α	214.63 ± 25.82	1079.74 ± 124.18^a^	188.03 ± 26.65	1268.01 ± 154.76^b^
IL-10	91.92 + 9.53	127.72 + 12.64^a^	98.76 + 6.82	72.35 + 5.47^a^

Cytokine concentrations were expressed as mean ± SEM in pg/mg protein; ^a^*P *<0.05 and ^b^*P *<0.01 versus matched controls (n = 8).

## Discussion

A summary and comparison of the results are presented in Table 3. According to these results the following several aspects are discussed.

**Table 3:** A summary of changes in glial cell markers, neurotrophins and cytokine concentrations in rat hippocampus after acute and subacute IL-1 administrations

	**Acute IL-1**		**Subacute IL-1**	
	**qPCR**	**Western or kit**	**qPCR**	**Western or kit**
CD11	NS	+	+	++
GFAP	NS	+	NS	_
NGF	+	+	NS	NS
BDNF	+	+	_	_
TrK A	NS	_	NS	+
TrK B	+	NS	_	_
p75	_	_	+	+
APP	NS	NS	+	+
IL-1β		NS		+
TNF-α		+		++
IL-10		+		_

As compared to acute and subacute IL-1 administrations with control groups, +, significantly up-regulated or increased; -, significantly down-regulated or decreased and NS, not significantly changed. GFAP, glial fibrillary acidic protein; NGF, nerve growth factor; BDNF, brain-derived neurotrophic factor; TrK, tyrosine kinase; APP, amyloid precursor protein.

### Acute and subacute IL-1 administration differently change glial activities, which may be related to inflammation in the brain

Acute and higher dose (100 ng/rat) i.c.v. administration of IL-1β has been found to activate microglia and astrocytes [33], which is similar to our findings in the present study. However, effects of chronic or subacute and low dose of IL-1 administrations on glial activities, which more accurately mirror the processes in neurodegenerative diseases, were not reported. Interestingly, the present study found that the increase in the protein expression of microglia marker CD11b was significantly greater after 8 days repeated IL-1 injections than after acute injections. Meanwhile, a more pronounced increase in the concentrations of pro-inflammatory cytokines was observed in the 8-day repeated IL-1 group than in the acute IL-1 group. Results from a mouse model of inducible sustained IL-1β overexpression bred with a triple transgenic mouse model of AD support our finding. In this model, greater microglial activation was found, which was also associated with inflammation [34]. On the other hand, changes in anti-inflammatory cytokine IL-10 and neurotrophins induced by acute and 8 days repeated IL-1 administration presented opposite effects. It is known that microglial, endothelial cells and astrocytes can produce anti-inflammatory cytokines, such as IL-10, while astrocytes can also produce neurotrophins [35,36]. In the present study, the up-regulation of GFAP expression and moderately increased CD11b expression were associated with increased IL-10 concentrations after acute IL-1 administration. However, a decrease in mRNA expressions of GFAP and a severe increase in CD 11b were found after 8 days repeated IL-1 administrations, which was associated with decreased IL-10. This result may indicate that astrocyte activation involves an anti-inflammatory response in acute neuroinflammation. Thus, the findings demonstrated the first part of our hypothesis that acute and subacute IL-1 administrations differentially modulate microglial and astrocyte activities, which may play different roles in acute and chronic neuroinflammation.

With regards to the role of astrocytes in neurodegeneration, there are currently two opposite opinions. Heneka and co-workers found that the reduction in activated microglia and several inflammatory responses in the brain were parallel with the decrease in Aβ1-42-positive amyloid deposits after treatments with the non-steroid anti-inflammatory drug ibuprofen [37]. However, ibuprofen also markedly inhibited reactive astrocytes in this APP transgenic model. These results support the hypothesis that astrocyte-induced inflammation contributes to neurodegenerative changes. However, it has been also reported that chronic ibuprofen administration worsens cognitive functions following traumatic brain injury in rats [38]. On the other hand, some evidence reveals that astrocytes have neuroprotective functions by inhibiting microglial activation and neuroinflammation in AD [39,40]. The results from the present study in glial cell activities and neurotrophin expression seems to support this later opinion since 8 days repeated IL-1 administration over-stimulated microglia but suppressed astrocyte functions, which resulted in the decrease of anti-inflammatory cytokines and BDNF neurotrophin and receptors.

There is a lack of parallel between CD11b mRNA and protein expressions. Because mRNA expressions are usually earlier than protein expressions, at a certain time point, their expression patterns could be at different stages.

### Acute and subacute IL-1 treatments induced opposite changes in the expressions of neurotrophins and their receptors

For the first time, the present study demonstrated that acute IL-1 injection markedly up-regulated mRNA and protein expressions of NGF and BDNF, while 8 days repeated IL-1 injection down-regulated BDNF mRNA and protein expression without significantly changing NGF expression and concentration in the hippocampus. Many studies have provided solid evidence that neurotrophins also act on mature neurons, particularly on injured and degenerated neurons. Studies in animal models of CNS diseases have shown that neurotrophins can reduce neuroinflammation and inhibit or delay neuronal death or degeneration [36,41]. In addition, increased NGF and BDNF expressions may contribute to the neuroinflammation-induced neuroprotection observed in acute brain injuries and early stages of stroke [21,42]. Conversely, the deficiency of BDNF or NGF is believed to be a cause or a trigger of neurodegeneration [43,44]. Thus, NGF and BDNF, or their receptor agonists, have been used to treat AD and other neurodegenerative diseases [12,45,46]. Dr O’Banion’s team has recently demonstrated that chronic over expression of IL-1 impairs adult hippocampal neurogenesis in the above mentioned mouse model [47]. Our data suggest that IL-1-induced dysfunction of neurotrophic system could be a contributor to the impairment of neurogenesis.

When compared to BDNF, NGF seems less sensitive to 8 days repeated IL-1 administration in the present study. A longer term of IL-1 administration may be needed in the future since we have previously reported that decreased NGF mRNA expressions were found in the hippocampus after IL-1 treatment for 8 days [5] or 10 days (unpublished results).

In AD patients and AD models, a decrease in the BDNF, its receptor, or its precursor form has been reported [48-50]. An increase in the BDNF concentration in the dentate gyrus of the hippocampus during exercise was associated with memory improvement in a neurodegeneration model of mice [51]. In the present study, the BDNF mRNA and protein expressions were up-regulated by acute IL-1 but down-regulated by 8 days repeated IL-1 administrations. Our results suggest that in response to acute brain insult or inflammation, both glial activities and neurotrophic functions are up-regulated, which may exert protection on the brain, while chronic inflammation, which increased microglia but suppressed astrocytes, may damage the neurotrophic system.

The functions of neurotrophin receptors and their relationship with neuroinflammation have also not been fully studied and understood in neurodegeration. As mentioned in the introduction, when neurotrophins combined with TrK receptors, cell survival should be promoted, while the activation of p75 receptors may trigger apoptosis. The present study demonstrated for the first time that acute IL-1 administration increased TrK B but reduced p75 receptor mRNA and protein expressions, which further supports the hypothesis that acute neuroinflammation may provide neuroprotective effects. Thus, our study demonstrated the second part of our hypothesis. However, it should be emphasized that the function of neurotrophin receptors in the brain is very complicated. For example, pro- and matured neurotrophins have been found to trigger different types of receptors [52]. Therefore, p75 receptors not only induce apoptosis but also modulate neurotrophin expressions and functions.

### The relationship between neurotrophins and their receptors and between neurotrophic systems and APP

Decreased TrK A receptors have been previously reported in the cortex of AD patients [53]. The present study found up-regulated TrK A protein expressions in the hippocampus associated with increased APP expressions, but decreased TrK A protein expression in the cortex (unpublished data) after subacute IL-1 injections, which may suggest that subacute IL-1 release in the brain could induce similar AD changes in this receptor.

A publication by D’Onofrio *et al*. [54] reported that in AD11 mice, an anti-NGF model (with no significant change in NGF mRNA expressions), the clusters of mRNAs that underwent the most changes were in the inflammatory category. The study also found that on postnatal day 30, when neurodegeneration and memory decline were undetectable, the mRNA expressions of BDNF were up-regulated, while on day 90, when Aβ deposition and memory decline started, the expressions of p75 and TrK A receptors were up-regulated but BDNF was down-regulated in the hippocampus. Another study has demonstrated that BDNF could inhibit Aβ production in primary neurons [55]. The present study demonstrated that after 8 days repeated IL-1 administration, an increase in the Aβ precursors APP expressions was associated with the decrease in BDNF mRNA and protein expressions and with the up-regulation of p75 and TrKA expressions, which provided strong evidence that IL-1β plays an important role in AD neuropathology.

As p75 receptors are a key ligand for Aβ, dysfunction of this receptor is linked with the neuropathology of AD; p75 can directly bind to Aβ1-42, leading to an increase in the secretion of APP, which produces Aβ and neurotoxicity [56], and conversely Aβ accumulation can stimulate p75 expressions [57]. In AD cortical neurons, the expression of p75 receptors is higher than in healthy controls [53]. In the present study, the increases in p75 mRNA and protein expressions were also accompanied by an increase in APP expressions in the hippocampus, while IL-1RA reversed the increase in p75 and also reversed APP expressions. Importantly, this change was only found after 8 days repeated, but not acute, IL-1 administrations. Thus, our hypothesis was further demonstrated.

### The relevance of IL-1β-induced changes to memory impairment in the neurotrophic system

Previously, Hein *et al*. [58] reported that in the mouse model with sustained hippocampal IL-1 overexpression, both spatial and contextual memory were impaired, which could have resulted from microglial activation and also from other increased inflammatory factors. The present study further explored microglia activation and inflammation-induced neurotrophin dysfunction. It is known that both NGF and BDNF have been found to enhance acetylcholine release [59,60], while deficiencies in acetylcholine release and BDNF or NGF expressions are related to memory impairment in AD patients, as mentioned in the introduction [5,49-51]. The results from the present study, in line with neurotrophin changes and inflammatory response, showed memory impairment 4 days after IL-1 administration. The rats treated with acute IL-1 did not show any memory impairment, as previously acute IL-1 administration has been found to enhance conditioned fear memory in rats [61,62]. The memory enhancement associated with acute IL-1 could be related to the up-regulated NGF, BDNF and TrK B expressions as observed in the present study. In this study, memory impairment was associated with down-regulated BDNF and TrK B and up-regulated TrK A and p75, and greater expression of p75 receptors in cholinergic neurons is believed to be responsible for cholinergic neuronal apoptosis and degeneration [63]. A previous study also reported that injecting IL-1β into the hippocampus could block the up-regulation of BDNF mRNA induced by conditioning memory [64]. The IL-1RA administration in the current study attenuated subacute IL-1-induced changes in BDNF, p75 and APP with significant improvement of memory performance in the radial maze. Therefore, the down-regulation of BDNF and up-regulation of p 75 may contribute to memory deficits in the radial maze after 8 days repeated IL-1 administration.

In summary, the present study demonstrated that acute and subacute IL-1 administration induced different changes in microglial and astrocyte activities, which cause opposite responses in neurotrophin expressions. Increased inflammatory responses, down-regulated BDNF and TrK B expressions, and up-regulated APP and p75 expressions may contribute to memory impairment after subacute or chronic IL-1 administration. However, results obtained from acute IL-1 administration, such as the up-regulation of NGF, BDNF and TrK B, increased anti-inflammatory cytokine IL-10 and down-regulated p75, may explain the neuroprotective functions of acute microglial and astrocyte activation and neuroinflammation in some brain conditions. Future studies should use glial inhibitors and anti-inflammatory mediators to further demonstrate each step of the pathway from glial activation to memory impairment.

## Abbreviations

ACh: Acetylcholine; AD: Alzheimer’s disease; ANOVA: Analysis of variance; APP: Amyloid precursor protein; BDNF: Brain-derived neurotrophic factor; CD11b: a microglial active marker; EDTA: Ethylenediamine-tetraacetic acid; ELISA: Enzyme-linked immunosorbent assay; GFAP: Glial fibrillary acidic protein; HRP: Horseradish peroxidase; IL: Interleukin; NGF: Nerve growth factor; p75: Low-affinity NGF receptor; PCR: Polymerase chain reaction; PMSF: Phenylmethylsulfonyl fluoride; RA: Receptor antagonist; SEM: Standard error of the mean; TMB: Tetramethyl benzidine; TNF: Tumor necrosis factor; TrK: Tyrosine kinase.

## Competing interests

The authors declare that they have no competing interests with respect to the authorship and/or publication of the article.

## Authors’ contributions

CS obtained the financial support, designed the experiment, trained researchers, analyzed data, prepared figures and tables and wrote the paper, YZ did a part of experiment (70%) and YD did a part of experiment (30%) and analyzed some of the data. All authors read and approved the final manuscript.
